# Reliability and Validity of the Perfusion, Extent, Depth, Infection and Sensation (PEDIS) Classification System and Score in Patients with Diabetic Foot Ulcer

**DOI:** 10.1371/journal.pone.0124739

**Published:** 2015-04-13

**Authors:** Fengning Chuan, Kang Tang, Peng Jiang, Bo Zhou, Xiaoqun He

**Affiliations:** Department of Endocrinology, the First Affiliated Hospital of Chongqing Medical University, Chongqing, China; Sapienza, University of Rome, School of Medicine and Psycology, ITALY

## Abstract

**Aims:**

To validate the perfusion, extent, depth, infection and sensation (PEDIS) classification system and to make the clinical practice easier, we created a score system and compared this system with two previously published common score systems.

**Methods:**

A retrospective cohort study was conducted on patients with diabetic foot ulcer (DFU) attending our hospital (n=364) from May 2007 to September 2013. Participants’ characteristics and all variables composing the PEDIS classification system were assessed.

**Results:**

During a median follow-up of 25 months (range 6-82), ulcers healed in 217 of the 364 patients (59.6%), remained unhealed in 37 patients (10.2%), and were resolved by amputation in 62 patients (17.0%); 48 patients (13.2%) died. When measured using the PEDIS classification system, the outcome of DFU deteriorated with increasing severity of each subcategory. Additionally, longer ulcer history, worse perfusion of lower limb, a larger extent of the ulcer, a deeper wound, more severe infection, and loss of protective sensation were independent predictors of adverse outcome. More importantly, the new PEDIS score system showed good diagnostic accuracy, especially when compared with the SINBAD and Wagner score systems.

**Conclusions:**

The PEDIS classification system, which encompasses relevant variables that contribute to the outcome of DFU and has excellent capacity for predicting the ulcer outcome, demonstrated acceptable accuracy. The PEDIS classification system might be useful in clinical practice and research both for the anticipation of health care costs and for comparing patient subgroups.

## Introduction

Diabetic foot ulcer (DFU) is a full-thickness wound, skin necrosis or gangrene below the ankle induced by peripheral neuropathy or peripheral arterial disease in patients with diabetes. It is one of the most common, severe and costly complications of diabetes and the most frequent cause for diabetes-associated hospitalization in China as well as the rest of the world [1, 2]. Because of diabetes-related delayed wound healing, DFU may lead to lower limb amputation, which deteriorates patients’ quality of life and increases mortality [3–5]. Given these various negative impacts, it is crucial to define a standardized and efficient approach to treat DFU in a timely manner; the first step should be the correct identification of degree of risk for ulcer-related complications in all patients with DFU [6].

Many DFU classification systems have been proposed to predict clinical outcome; however, almost of these systems have limitations. First, the majority of the classification systems only focus on local pathology of DFU and fail to adequately assess all the important parameters related to ulcer healing. For example, the Wagner system exclusively assesses ulcer depth without co-morbidities such as ischemia and neuropathy [7]. Second, few classification systems incorporate standardized definitions of ischemia, infection and systemic variables important to wound healing. Finally, few classification systems of DFU have been validated, and no classification has gained universal acceptance [8]. To categorize and define DFU objectively and facilitate communication between health-care providers, the International Working Group of the Diabetic Foot (IWGDF) developed the Perfusion, Extent, Depth, Infection and Sensation (PEDIS) classification system in which all DFUs are classified according to five categories: perfusion, extent/size, depth/tissue loss, infection and sensation. These categories were considered to be the most relevant pathogenesis of the development of DFU. Moreover, each subcategory is defined according to strict criteria based upon objective techniques, which are applicable worldwide [9]. The PEDIS classification system was developed primarily for research and has not yet been validated in clinical practice regarding prognosis [8].

So it was hypothesized that the PEDIS classification system is more objective and exact to assess DFU to predict the clinical outcome than previous systems such as Wagner system and the Site, Ischemia, Neuropathy, Bacterial, Infection, and Depth (SINBAD) system, because the PEDIS classification system defined DFU according to more strict criteria based upon objective techniques and more comprehensive parameters related to ulcer healing were considered. Therefore, the aims of this study were to evaluate the reliability and the accuracy of the PEDIS classification system, create a score system based on the PEDIS system to facilitate its use in clinical practice, and compare the new score system to two previously published common score systems.

## Methods

### Type of study and selection of participants

A retrospective cohort study was conducted in the First Teaching Hospital of Chongqing Medical University, a tertiary care setting with a multidisciplinary foot care team. The study took place between May 2007 and September 2013 and involved a total of 364 inpatients with DFU. The inclusion criteria were patients with type 1/2 diabetes having at least 1 foot ulcer; if more than 1 foot ulcer was present, the ulcer most recently identified was selected as the index ulcer. If two or more ulcers were registered at the same time, the one that was judged to be most significant was chosen [6]. We excluded patients with secondary diabetes, foot ulcers caused by autoimmune disease or malignancies, and foot ulcers in association with acute foot ischemia [10]. Written informed consent was obtained from all participants. The study was approved by the Ethics Committee of the first affiliated hospital of Chongqing medical university, Chongqing, China.

### Data collection

We obtained basic demographic data for these patients using structured interviews and electronic medical records including age, sex, body mass index (BMI), smoking history, alcohol intake, type of diabetes, treatment and duration of diabetes, and ulcer history (defined as the time elapsed between the onset of symptoms and hospital admission). In addition, we collected levels of hemoglobin (Hb), serum albumin, fasting plasma glucose (FPG), glycated hemoglobin (HbA_1c_), white blood cells (WBC), estimated glomerular filtration rate (eGFR) [11, 12], total cholesterol (TC), triacylglycerol (TG), low density lipoprotein cholesterol (LDL-C), and high density lipoprotein cholesterol (HDL-C) from the clinical records. Also noted were complications [13] associated with diabetes such as peripheral arterial disease, retinopathy, nephropathy, peripheral neuropathy, and autonomic neuropathy as well as other co-morbidities such as hypertension, coronary heart disease, and stroke, which were diagnosed by the treating physicians and documented in the medical records.

### Classification

We classified all DFUs according to the five categories: perfusion, extent, depth, infection and sensation based on the PEDIS classification system. 1) Perfusion was determined by a combination of physical examination and noninvasive vascular studies. Clinical signs were based on the absence of dorsal pedal or posterior tibial artery pulses of the involved foot. Noninvasive criteria included the ankle-brachial index (ABI), toe-brachial index (TBI), transcutaneous oxygen pressure (TcpO_2_) and ankle/toe pressure. 2) Extent was estimated by multiplying the largest diameter by the second largest diameter measured perpendicular to the first diameter and expressed as cm^2^. To standardize the score of the extent category, we allocated ulcers to one of the following groups as performed previously [6]: skin intact, <1 cm^2^, 1–3 cm^2^, or >3 cm^2^. 3) Depth was evaluated using a sterile blunt nasal probe and imaging tests. 4) The diagnosis of infection was based principally on the presence or absence of symptoms and signs of inflammation, and the presence of secretion, the results of laboratory tests and imaging tests. 5) Sensation was evaluated with a 10-g monofilament and/or a 128-Hz tuning fork sensation on one or more sites of the foot [9].

### Score

To facilitate use of the PEDIS classification system by clinicians, we created the PEDIS score system. The details of the PEDIS score system are shown in Table 1. Overall score is determined by adding the five separate categories to a theoretical maximum of 12. The SINBAD and Wagner score systems are already widely accepted, and both classify DFU mainly on clinical measures. In the SINBAD system [14], these six elements are graded as follows: 1) ulcer site (forefoot, 0, and midfoot/hindfoot, 1); 2) ischemia (blood flow relatively intact, 0, and evidence of ischemia, 1); 3) neuropathy, defined as being absent, 0, or present, 1, on the basis of routine examination using 10-g monofilaments; 4) bacterial infection (using clinical signs of infection of either soft tissue or bone proposed by the Infectious Diseases Society of America and the IWGDF), graded as absent, 0, or present, 1; 5) area (the two maximum dimensions at right angles multiplied [<1 cm^2^, 0, and >1 cm^2^, 1]); and 6) depth (superficial, 0, and deep [reaching to tendon, periosteum, joint capsule, or bone], 1). In addition, the individual grades were summed, creating a SINBAD score range of 0–6. The Wagner system [8] comprises a 5-point scale: score 0 (intact skin), score 1 (superficial ulcer), score 2 (deep ulcer to tendon, bone or joint), score 3 (deep ulcer with abscess or osteomyelitis), score 4 (forefoot gangrene) and score 5 (whole foot gangrene).

**Table 1:** The PEDIS classification system and the score system.

Grade	Perfusion	Extent	Depth	Infection	Sensation	Score
1	No PAD	Skin intact	Skin intact	None	No loss	0
2	PAD, No CLI	<1 cm^2^	Superficial	Surface	Loss	1
3	CLI	1–3 cm^2^	Fascia, muscle, tendon	Abscess, fasciitis, septic arthritis		2
4		>3 cm^2^	Bone or joint	SIRS		3

PAD, peripheral arterial disease; CLI, critical limb ischemia.

### Outcome

All patients were followed for at least 6 months or until death. Outcome [4, 15] was categorized as healed (defined as a continuous, viable epithelial covering over the entire previously open wound), unhealed (defined as not complete re-epithelialization of the wound), amputation (included minor and major amputations) or death (whether from related causes of DFU or not). For the purpose of cross-tabulation, correlation with baseline variables and logistic regression analysis, the outcomes were regrouped as either desired outcome (healed) or adverse outcome (unhealed, amputation or death).

### Statistical analysis

A descriptive analysis was conducted to assess the characteristics of the samples. Categorical data were expressed as numbers, and a chi-squared test or Fisher’s exact test was used to evaluate the distribution differences. The normal distribution continuous variables were expressed as the mean ± standard deviation (SD); abnormal distribution variables were expressed as the interquartile range, and normalized using logarithmic transformation. The differences were tested with the one-way ANOVA. A chi-squared test for trend was used to assess the trend association between increasing grade of PEDIS classification and the prevalence of adverse outcome. A multivariable logistic regression analysis was performed to assess which characteristics and clinical variables were independently associated with outcome using variables with P<0.05 according to the univariate analysis. The receiver operating characteristic (ROC) curves of the PEDIS score system were used to determine optimal threshold values in which the point-of-care device could help clinicians to predict the outcome of DFU. Additionally, the ROC curves were also made with Wagner and SINBAD score systems and compared with the PEDIS score system. We conducted all analyses using SPSS version 20.0 statistical software (SPSS, Chicago, IL, USA); P<0.05 was considered to be statistically significant.

## Results

### Description of participants

This study included 364 patients with a median follow-up of 25 months (range 6–82). Of all ulcers, 219 were in male subjects (60.2%) and 145 were in female subjects (39.8%). Median age was 66 years (23–95), and the mean diabetes duration was 9.9 years (0.25–50). Median ulcer history was 35 days (1–240). Of all patients, ulcers healed in 217 of the 364 patients (59.6%), while 62 (17.0%) had been resolved by amputation; 48 patients (13.2%) died. Ulcers of 37 patients (10.2%) persisted unhealed on the day of observation (Table 2).

**Table 2:** Comparison of all the characteristics with the different outcomes.

Parameter	All	Healed	Unhealed	Amputated	Death	P†	P‡
**Demographics**							
Gender (male/female)	219/145	125/92	28/9	38/24	28/20	0.221	
Age (years)	66.3±11.66	64.95±11.30	64.78±13.20	68.48±11.36	70.9±11.13	0.004	0.034^b^
BMI (kg/m^2^)	22.26±3.01	22.53±2.94	21.79±2.33	22.12±3.69	21.56±2.69	0.144	
Smoking habits (yes/no)	116/248	64/153	16/21	24/38	12/36	0.161	
Alcohol misuse (yes/no)	101/263	58/159	12/25	21/41	10/38	0.420	
**Diabetic history**							
Diabetes type (Type 2/Type 1)	358/6	214/3	36/1	60/2	48/0	0.863	
Diabetic duration (years)	9.88±7.46	9.47±7.02	11.03±9.44	10.44±6.95	10.09±8.33	0.594	
Ulcer history (days)	34.9±41.33	22.52±27.42	34.95±32.40	66.42±58.12	50.7±47.69	<0.001	0.000^b^
							0.000^c^
Treatment: oral drugs/insulin	156/208	88/129	21/16	26/36	21/27	0.330	
**Laboratory test**							
Fasting plasma glucose (mmol/l)	11.56±2.74	11.33±2.80	12.37±2.55	11.56±2.53	12.00±2.81	0.110	
HbA_1c_(%)	8.83±1.62	8.83±1.61	9.21±1.86	8.67±1.61	8.71±1.45	0.404	
HbA_1c_ (mmol/mol)	73±18	73±17	77±21	71±18	72±16	0.404	
White blood cells (10^3^/ul)	8.73±4.63	7.92±3.73	8.72±4.71	9.99±4.64	10.79±6.96	<0.001	0.002^b^
							0.000^c^
Hemoglobin(g/l)	113.90±21.30	117.00±20.86	108.49±23.96	115.18±19.33	102.45±19.39	<0.001	0.022^a^
							0.000^c^
Serum albumin (g/l)	34.00±6.25	35.47±5.60	33.36±5.67	31.98±6.89	30.38±6.48	<0.001	0.050^a^
							0.000^b^
							0.000^c^
eGFR (ml/min 1.73 m^2^)	87.79±33.74	89.29±31.97	85.82±34.91	90.67±34.12	79.58±38.94	0.298	
Total cholesterol (mmol/l)	4.01±0.65	4.05±0.64	4.05±0.71	3.96±0.63	3.90±0.69	0.460	
LDL cholesterol (mmol/l)	2.23±0.62	2.29±0.57	2.21±0.66	2.13±0.61	2.11±0.76	0.123	
Triglycerides (mmol/l)	1.38±0.45	1.39±0.47	1.34±0.43	1.35±0.42	1.36±0.42	0.906	
HDL (mmol/l)	1.11±0.23	1.13±0.25	1.06±0.21	1.11±0.18	1.11±0.19	0.308	
**Co-morbidities**							
Hypertension (yes/no)	179/185	106/111	12/25	36/26	25/23	0.099	
Cardiac heart disease (yes/no)	61/303	36/181	3/34	9/53	13/35	0.118	
History of stroke (yes/no)	47/317	26/191	6/31	9/53	6/42	0.879	
**Diabetic complication**							
Nephropathy (yes/no)	156/208	96/121	15/22	21/41	24/24	0.348	
Retinopathy (yes/no)	107/257	71/146	10/27	14/48	12/36	0.378	
Peripheral arterial disease (yes/no)	207/157	107/110	25/12	44/18	31/17	0.005	0.003^b^
Peripheral neuropathy (yes/no)	283/81	172/45	29/8	44/18	38/10	0.572	
Autonomic neuropathy (yes/no)	18/364	10/207	3/34	3/59	2/46	0.895	

eGFR, estimated glomerular filtration rate.

^†^overall differences between groups;

^‡^significant difference from groups.

^a^: between healed and unhealed;

^b^: between healed and amputated;

^c^: between healed and death.

### Outcome of PEDIS classification system

The outcomes of different subcategories of the PEDIS classification system are summarized in Table 3. With increasing severity of each subcategory of PEDIS classification system, there was a statistically significant trend toward an increased risk for adverse outcome. This correlation indicates that those patients with adverse outcome had worse perfusion of lower limb, larger extent of the ulcer, deeper ulcer, more severe infection or loss of protective sensation (Table 3).

**Table 3:** Outcome of different grades of PEDIS classification system.

Variable = grade	Healed (%)	Unhealed (%)	Amputated (%)	Death (%)
Perfusion^a^ = 1	153 (83.6%)	15 (8.2%)	13 (7.1%)	2 (1.1%)
Perfusion^a^ = 2	61 (42.1%)	21 (14.5%)	33 (22.8%)	30 (20.7%)
Perfusion^a^ = 3	3 (8.3%)	1 (2.8%)	16 (44.4%)	16 (44.4%)
Extent^a^ = 1	94 (83.9%)	6 (5.4%)	10 (8.9%)	2 (1.8%)
Extent^a^ = 2	75 (58.1%)	16 (12.4%)	20(15.5%)	18 (14.0%)
Extent^a^ = 3	48 (39.0%)	15 (12.2%)	32 (26.0%)	28 (22.8%)
Depth^a^ = 1	78 (97.5%)	0 (0.0%)	2 (2.5%)	0 (0.0%)
Depth^a^ = 2	131 (57.7%)	27(11.9%)	41(18.1%)	28 (12.3%)
Depth^a^ = 3	8 (14.0%)	10 (17.5%)	19 (33.3%)	20 (35.1%)
Infection^a^ = 1	15 (100.0%)	0 (0.0%)	0 (0.0%)	0 (0.0%)
Infection^a^ = 2	175 (78.8%)	18 (8.1%)	18 (8.1%)	11 (5.0%)
Infection^a^ = 3	25 (25.3%)	16 (16.2%)	39 (39.4%)	19 (19.2%)
Infection^a^ = 4	2 (7.1%)	3 (10.7%)	5 (17.9%)	18 (64.3%)
Sensation^a^ = 1	125 (94.7%)	7 (5.3%)	0 (0.0%)	0 (0.0%)
Sensation^a^ = 2	92 (39.7%)	30 (12.9%)	62 (26.7%)	48 (20.7%)

^a^ χ^2^ Score for trend, P<0.05

### Association of variables with DFU outcome

The patients with adverse outcomes were more likely to be older, have a longer ulcer history, have lower levels of Hb and serum albumin, and have a higher level of WBC level compared to patients with desired outcomes. Additionally, patients with adverse outcomes tended to have coexisting conditions more frequently than those with the desire outcome, including peripheral arterial disease.

To examine the independent effects on outcome, logistic regression analysis was performed with outcome (desired outcome or adverse outcome) as the dependent variable and baseline categories as independent variables. The independent variables that contributed significantly to the model were longer ulcer history (OR, 1.024, 95% CI, 1.010–1.039, P = 0.001), worse perfusion of lower limb (OR, 8.098, 95% CI, 3.658–17.929, P<0.001), larger extent of the ulcer (OR, 2.461, 95% CI, 1.373–4.412, P = 0.002), deeper wound (OR, 12.494, 95% CI, 4.076–38.297, P<0.001), more severe infection (OR, 7.202, 95% CI, 3.407–15.224, P<0.001), and loss of protective sensation (OR, 9.545, 95% CI, 3.184–28.611, P<0.001).

### Accuracy of the PEDIS score system

In addition, the five separate categories of PEDIS classification system were summed, creating a PEDIS score range of 0–12 (Table 1). Subsequently, we wanted to explore the existence of a distinct threshold value that could help clinicians to predict the outcome of DFU. The threshold value that maximized sensitivity and specificity for adverse outcomes based on the ROC curve analysis was 7. For identification of adverse outcomes, the ROC curve had an area under the curve (AUC) of 0.95 and the threshold value had a sensitivity of 93% and a specificity of 82%. In comparison, the ROC curves of the SINBAD and Wagner score systems had an AUC of 0.88 and 0.86, respectively, and the threshold value had a sensitivity of 90% and 88%, respectively, and a specificity of 73% and 80%, respectively (S1 Fig).

## Discussion

A validated classification system of DFU may help clinicians in everyday assessment and management of patients as well as researchers in the development and assessment of new therapies [8]. To our knowledge, this is the first time that the PEDIS classification system was validated to predict clinical outcome. The results of this study indicate that both the category and grade affect the outcome independently, and the higher the grade of subcategory, the greater the chance that the ulcer will persist or that death will occur. The most important finding of this study is that the simple PEDIS score system can also predict the outcome and may be more accurate than the more widely used systems.

DFUs represent a heterogeneous pathological entity, caused by a broad range of etiological factors in a diverse patient population [1]. Lawrence *et al*. [16] demonstrated the relationship between infection severity and the necessity for amputation, but other factors related to adverse outcome were not included. Additionally, Oyibo *et al*. [17] and other previous studies [18–20] have confirmed the outcome of DFUs to be influenced by blood supply, presence of infection, depth of ulcers and area of ulcers, but they were not validated independently. The PEDIS classification system contains five similar categories highlighting their clinical relevance, and our data support that the observation that increased severity of each subcategory correlates with worse outcomes of DFU. Additionally, with multivariate analysis, our data further showed that all five categories had an independent impact on the outcome of DFU. The trend of the grade and the independence of these factors support the clinical value of the PEDIS classification system in predicting clinical outcomes.

Many score systems have been proposed with the purpose of facilitating quick and accurate clinical decisions. Monteiro *et al*. [21] used ROC curve analysis to assess the different systems’ diagnostic accuracy for DFU development and considered it to be the best method for determining a system’s discriminatory ability [22, 23]. In addition to ROC curve analysis, we also used the AUC value to confirm the diagnostic accuracy of the PEDIS score system to predict the outcome of DFUs. The results of this study indicate that the PEDIS score system also has excellent capacity to predict the outcome. In addition, our study shows that the PEDIS category scores can be summed into an aggregate PEDIS score, with a score of 7 or more being associated with a significantly greater probability of difficulties in healing. We believe that the PEDIS score system should be applied widely in clinical practice.

The intra-rater and inter-rater reliability of variables are difficult to perform due to the retrospective character of this study as well as the dynamic nature of ulcer characteristics [6]. Because the PEDIS classification system includes strict definitions and categorization based on objective techniques such as TcpO_2_ [24] that are applicable worldwide, the system has the potential for broad acceptance. When comparing the PEDIS score system with two commonly used score systems, SINBAD and Wagner, the PEDIS score system had the highest AUC value and therefore most accurately predicted adverse outcomes. In addition, the PEDIS score system presented the highest sensitivity, specificity and positive likelihood ratios (LR) as well as the lowest negative LR for adverse outcomes. Regarding the Wagner and SINBAD score systems [7, 14], classifications of DFUs are based mainly on clinical methods, and the parameters of these systems are not sufficiently comprehensive or accurate to assess the features and severity of ulcers.

In conclusion, the study showed that patients with DFU who had a longer ulcer history, worse perfusion of the lower limb, a larger extent (surface area) of the ulcer, a deeper wound, more severe infection, and loss of protective sensation will be less likely to heal. Persistent ulcers likely have a significant impact on the use of resources in this population. Therefore, in clinical practice, medical personnel should pay close attention to and actively evaluate these indexes, which may contribute to better and more standardized treatment of DFUs.

### Limitations

First, the data set used in this study was generated from one hospital, limiting its generalizability to other hospitals; therefore, further validation studies should be performed on larger samples and in different settings with a longer follow-up period. In addition, retrospective surveys have inherent deficiencies; a prospective design would be preferable to establish the direction of causality.

## Supporting Information

S1 Fig ROC curve showing the accuracy of PEDIS score system, compare with SINBAD and Wagner system. Straight line, PEDIS score system; short dashed line, SINBAD system; long dashed line, Wagner system. An optimal threshold of PEDIS score system for adverse outcome had a sensitivity of 93% and a specificity of 82%. In comparison, the threshold value of SINBAD and Wagner system had a sensitivity of 90% and 88%, respectively, and a specificity of 73% and 80%, respectively.
(TIF)
