# Supplementary figures and images for: Reliability and Validity of the Perfusion, Extent, Depth, Infection and Sensation (PEDIS) Classification System and Score in Patients with Diabetic Foot Ulcer
Source: PLoS One. 2015 Apr 13;10(4):e0124739. doi: 10.1371/journal.pone.0124739 (PMC4395335; doi:10.1371/journal.pone.0124739)

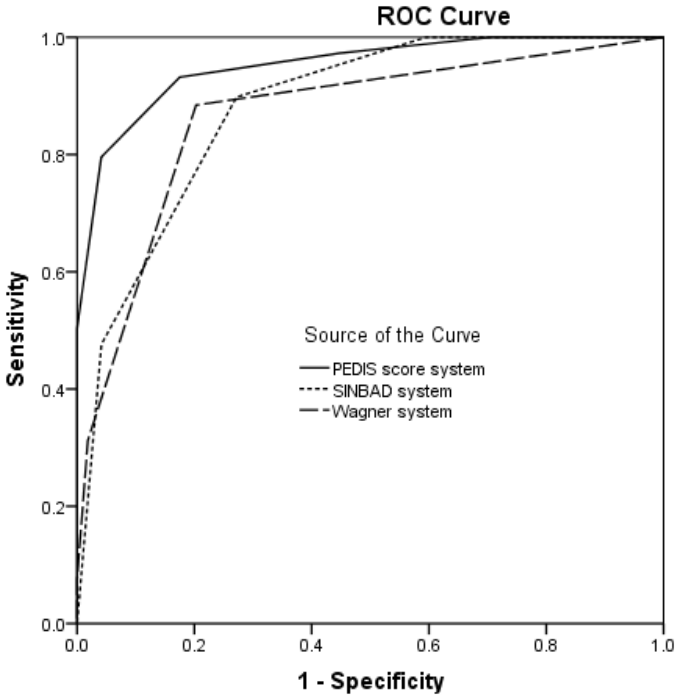

Supplement: S1 Fig — Straight line, PEDIS score system; short dashed line, SINBAD system; long dashed line, Wagner system. An optimal threshold of PEDIS score system for adverse outcome had a sensitivity of 93% and a specificity of 82%. In comparison, the threshold value of SINBAD and Wagner system had a sensitivity of 90% and 88%, respectively, and a specificity of 73% and 80%, respectively. (TIF) [file pone.0124739.s001.tif]
